# Loss of AMP-Activated Protein Kinase Induces Mitochondrial Dysfunction and Proinflammatory Response in Unstimulated Abcd1-Knockout Mice Mixed Glial Cells

**DOI:** 10.1155/2015/176983

**Published:** 2015-03-15

**Authors:** Jaspreet Singh, Hamid Suhail, Shailendra Giri

**Affiliations:** Department of Neurology, Henry Ford Health System, Detroit, MI 48202, USA

## Abstract

X-linked adrenoleukodystrophy (X-ALD) is caused by mutations and/or deletions in the ABCD1 gene. Similar mutations/deletions can give rise to variable phenotypes ranging from mild adrenomyeloneuropathy (AMN) to inflammatory fatal cerebral adrenoleukodystrophy (ALD) via unknown mechanisms. We recently reported the loss of the anti-inflammatory protein adenosine monophosphate activated protein kinase (AMPK*α*1) exclusively in ALD patient-derived cells. X-ALD mouse model (Abcd1-knockout (KO) mice) mimics the human AMN phenotype and does not develop the cerebral inflammation characteristic of human ALD. In this study we document that AMPK*α*1 levels *in vivo* (in brain cortex and spinal cord) and *in vitro* in Abcd1-KO mixed glial cells are similar to that of wild type mice. Deletion of AMPK*α*1 in the mixed glial cells of Abcd1-KO mice induced spontaneous mitochondrial dysfunction (lower oxygen consumption rate and ATP levels). Mitochondrial dysfunction in ALD patient-derived cells and in AMPK*α*1-deleted Abcd1-KO mice mixed glial cells was accompanied by lower levels of mitochondrial complex (1-V) subunits. More importantly, AMPK*α*1 deletion induced proinflammatory inducible nitric oxide synthase levels in the unstimulated Abcd1-KO mice mixed glial cells. Taken together, this study provides novel direct evidence for a causal role for AMPK loss in the development of mitochondrial dysfunction and proinflammatory response in X-ALD.

## 1. Introduction

X-linked adrenoleukodystrophy (X-ALD) is an inherited neuroinflammatory demyelinating peroxisomal disorder [1]. The underlying defect is a mutation/deletion in the ABCD1 gene that encodes the peroxisomal integral membrane transporter adrenoleukodystrophy protein (ALDP) [2]. ALDP is responsible for importing very long chain fatty acids (VLCFA; C >22 : 0) into the peroxisomes for degradation, a function exclusive to peroxisomes. As a result, VLCFA accumulate in the tissues and body fluids of X-ALD patients, a biochemical hallmark of the disease [3]. The disease has two major phenotypes: severe inflammatory and often fatal cerebral adrenoleukodystrophy (ALD) and mild relatively benign adrenomyeloneuropathy (AMN) [1, 3]. ALD patients develop spontaneous neuroinflammatory responses and demyelination, which results in death within 2–5 years from the onset of symptoms [1]. AMN patients, on the other hand, live into adulthood with mild axonopathy [1]. However, about 30% of AMN patients progress spontaneously to fatal ALD phenotype in adulthood [1]. The mechanism(s) for differential phenotypes (AMN or ALD) or the progress of AMN to ALD phenotype remain unknown [1, 4]. Intriguingly, the ABCD1 mutation and VLCFA levels are common among the two major phenotypes [4]. In fact, both the phenotypes are detectable within a family with similar ABCD1 mutations; thus, there is no phenotype-genotype correlation [1]. An animal model of X-ALD, a classical knockout of Abcd1 (Abcd1-KO), accumulates VLCFA in tissues and body fluids but fails to develop the neuroinflammatory response [5–7]. In the late stage of life (>15 months), the mice develop axonopathy in the spinal cord and thus the mouse model at best resembles the human AMN phenotype [8].

We recently documented the first evidence of loss of a metabolic gene, AMP-activated protein kinase (AMPK), in ALD but not AMN patient-derived cells [9].* In vivo* and* in vitro* studies have shown that AMPK signaling and proinflammatory responses are mutually coupled via negative feedback [10–12]. Activation of AMPK suppresses proinflammatory mediators [13, 14] while stimulation with inflammatory cytokines promotes dephosphorylation and hence inhibition of AMPK [14]. ALD patient-derived cells lacking AMPK demonstrated an increased proinflammatory gene expression [9]. Mitochondrial dysfunction (measured as oxygen consumption rate (OCR)) was also observed in ALD patient-derived cells [9]. This was not surprising considering that AMPK is the principal upstream regulator of mitochondrial function and loss of AMPK induces spontaneous mitochondrial dysfunction and proinflammatory response both* in vivo* and* in vitro* [12, 15]. A direct causal role for AMPK*α*1 in the X-ALD neuroinflammatory response, however, remained to be investigated. The status of AMPK*α*1 in Abcd1-KO mice central nervous systems is unknown. Since Abcd1-KO mice mimic the human AMN phenotype [5–7] and do not develop the cerebral inflammation characteristic of human ALD [5–8], in this study we investigated the status of AMPK*α*1 in the brains and spinal cords of Abcd1-KO mice. Furthermore, the expression and levels of AMPK*α*1, mitochondrial complex subunits, and mitochondrial OCR were compared between wild type (WT) and Abcd1-KO mice mixed glial cells. To investigate a causal role for AMPK*α*1 in the development of the neuroinflammatory response in X-ALD, we used lentiviral vector carrying mouse AMPK*α*1-shRNA to delete AMPK*α*1 in Abcd1-KO mouse primary mixed glial cells. Mitochondrial function (OCR) and induction of proinflammatory response were compared between WT, Abcd1-KO, and AMPK*α*1-deleted Abcd1-KO mice mixed glial cells.

## 2. Materials and Methods

### 2.1. Reagents

Dulbecco's Modified Eagle's Medium (DMEM, 4.5 g/L) and fetal bovine serum were purchased from BioAbChem Inc. (Ladson, SC). Antibodies were purchased from Cell Signaling Technology Inc. (Boston, MA), unless otherwise mentioned. ECL and nitrocellulose membranes were purchased from BioRad (Hercules, CA). MitoProfile Total OXPHOS WB Antibody Cocktail was purchased from Abcam (Cambridge, MA). ATP determination kit was from Molecular Probes (Invitrogen, Grand Island, NY).

### 2.2. Abcd1-Knockout (Abcd1-KO) Mice

Abcd1-KO mice were obtained from Jackson Laboratories and housed in the pathogen-free animal housing facility of the Henry Ford Health System. Animal procedure (#1271) was approved by the Henry Ford Health System Animal Review Committee, and all animals received humane care in compliance with the institution's experimental guidelines (Guide for the Care and Use of Laboratory Animals).

### 2.3. Cell Culture

#### 2.3.1. Fibroblasts

Human skin fibroblasts derived from healthy control (CTL1, GM03348, CTL2, and GM03377), ALD (ALD1, GM04934, ALD2, and GM04904), and AMN (AMN1, GM07531, AMN2, and GM17819) patients were obtained from the National Institute of General Medical Sciences Human Genetic Cell Repository (https://catalog.coriell.org/) and cultured as described previously [9].

#### 2.3.2. Primary Mixed Glial Cells

Mouse primary mixed glial cells were prepared from 2-day-old WT and Abcd1-KO pups, as described previously [16].

### 2.4. Lentiviral Vector Mediated Knockdown of AMPK*α*1 in Abcd1-KO Mice Mixed Glial Cells

Transduction-ready mouse shRNA lentiviral particles (10^6^ TU/mL) for AMPK*α*1 (consisting of a pool of 3–5 constructs and puromycin selection gene; sc-29674-V) and control shRNA lentiviral particles (Scr) (10^6^ TU/mL, sc-108080) were purchased from Santa Cruz Biotechnology (Dallas, TX).

Abcd1-KO mixed glial cells were cultured in DMEM with 10% fetal bovine serum, and viral particles (AMPK*α*1 and control) were added with a multiplicity of infection of 2.5. Transduced cells were selected using puromycin (3.0 *μ*g/mL). AMPK*α*1 silencing was observed by western blot and mRNA quantification.

### 2.5. Western Blot Analysis

Samples for western blot were prepared and ran as described previously [9]. The membranes were probed with AMPK*α*1, PGC-1*α* (Santa Cruz Biotechnology, Dallas, TX), or MitoProfile Total OXPHOS Rodent WB Antibody Cocktail. The membranes were detected by autoradiography using ECL-plus.

### 2.6. RNA Extraction and cDNA Synthesis

Following total RNA extraction using TRIzol (Invitrogen), per the manufacturer's protocol, single-stranded cDNA was synthesized from 5 *μ*g of total RNA using iScript cDNA synthesis kit (BioRad, Hercules, CA).

### 2.7. Real-Time Polymerase Chain Reaction

Real-time polymerase chain reaction (PCR) was conducted using Bio-Rad's CFX96 Real-Time PCR Detection System. The primer sets for use were synthesized from Integrated DNA Technologies (Coralville, IA). IQ SYBR Green Supermix was purchased from Bio-Rad. The normalized expression of target gene with respect to L27 was computed for all samples.

### 2.8. Measurement of Mitochondrial Oxygen Consumption and Extracellular Flux

Oxygen consumption in intact adherent WT and Abcd1-KO mixed glial cells was measured using a Seahorse Bioscience XF^e^96 Extracellular Flux Analyzer (North Billerica, MA), as previously described [9]. Mixed glial cells were seeded to 1.5 × 10^4^ cells/well in XF^e^96-well cell culture microplate (Seahorse Bioscience) in 200 *μ*L of DMEM and cultured at 37°C in 5% CO_2_ atmosphere. The growth medium was replaced with 175 *μ*L of bicarbonate-free DMEM, and the cells were incubated for 1 hour for degassing before starting the assay procedure. Basal and carbonyl cyanide p-trifluoromethoxyphenylhydrazone- (FCCP-) linked OCR was measured as described by us previously [9].

### 2.9. Determination of ATP Levels

Primary Abcd1-KO mice mixed glial cells (2 × 10^4^ cells/well) were seeded in a 96-well cell culture plate in complete medium and deleted for AMPK*α*1 as described above. Cells were lysed in 20 *μ*L lysis buffer and 10 *μ*L of lysate was used to measure ATP levels using an ATP determination kit (Molecular Probes, Invitrogen). 1 *μ*L of the cell lysate was used for normalization of protein levels.

### 2.10. Statistical Analysis

Using the Student Newman-Keuls test and analysis of variance, *P* values were determined for the respective experiments from three identical experiments using GraphPad software (GraphPad Software Inc., San Diego, CA). The criterion for statistical significance was *P* < 0.05.

## 3. Result and Discussion

### 3.1. AMPK*α*1 Levels Are Similar between WT and Abcd1-KO Mice Brains and Mixed Glial Cells

**Figure 1:**
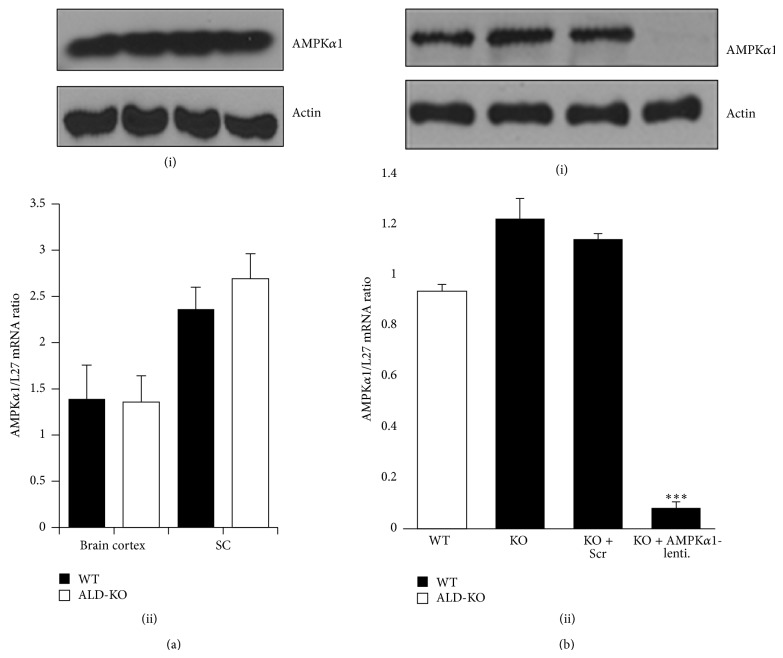
Adenosine monophosphate activated protein kinase (AMPK*α*1) levels are similar* in vivo* in wild type (WT) and Abcd1-knockout (KO) central nervous systems and* in vitro* in mixed glial cells. (a) Age-matched (3-month-old) WT and Abcd1-KO mice were sacrificed and the brains and spinal cords harvested for AMPK*α*1 protein (i) and mRNA (ii) levels. (b) In primary mixed glial cells from WT and Abcd1-KO mice, AMPK*α*1 protein (i) and mRNA (ii) levels were similar. (b) Abcd1-KO mixed glial cells were silenced for scrambled control (Scr) or AMPK*α*1 as described in Section 2. Lentiviral vector silencing with AMPK*α*1-shRNA significantly decreased the AMPK*α*1 protein (i) and mRNA (ii) levels in Abcd1-KO mice primary mixed glial cells. Results represent the mean ± SD of triplicates from three different experiments. ^***^ *P* < 0.001 AMPK*α*1 silenced compared with scrambled (Scr) silenced.

The mechanism(s) of induction of the neuroinflammatory response in X-ALD remains unknown [1, 4]. More intriguing is the observation that the inflammatory response and demyelination have no genotype correlation [1, 4]. Individuals with the same ABCD1 mutation (even between monozygotic twins) may develop strikingly opposite phenotypes of fatal neuroinflammatory ALD (and hence a shortened life span) or relatively benign AMN phenotype that exhibits only mild spinal cord pathology that is too late in adulthood [1, 3]. We recently documented the first evidence of differential loss of a metabolic and anti-inflammatory protein, AMPK*α*1, in the patient-derived fibroblasts and lymphocytes of the severe (ALD) phenotype of X-ALD [9]. AMPK*α*1 levels between healthy control and AMN patient-derived cells were largely unchanged [9]. Our laboratory previously reported that loss of AMPK*α*1 is associated with more severe neuroinflammation and neurodegeneration in an animal model of multiple sclerosis [17]. Abcd1-KO mice fail to develop the neuroinflammation and demyelination characteristic of human ALD and only mimic the human mild AMN phenotype [5–8]. It was, therefore, of interest to investigate the status of AMPK*α*1 in Abcd1-KO mice brains and spinal cords. In line with our recent report in human AMN patient-derived cells (compared to healthy controls) [9], there was no significant difference in AMPK*α*1 protein and mRNA levels in the brain cortexes or spinal cord of age-matched (3-month-old) Abcd1-KO mice compared with WT mice (Figure 1(a)).

Also, AMPK*α*1 levels in the primary mixed glial cells of Abcd1-KO mice were not decreased and were, in fact, higher than those in WT mice mixed glial cells (Figure 1(b)). In both cells and animal models with AMPK deletion, there is spontaneous mitochondrial dysfunction and proinflammatory response [10, 12, 18, 19]. To investigate if AMPK*α*1 loss in Abcd1-KO mice mixed glial cells can induce mitochondrial dysfunction and inflammation reminiscent of the ALD phenotype, Abcd1-KO mixed glial cells were silenced for AMPK*α*1 using lentiviral-shRNA (Figure 1(b)). Following transduction and selection of transduced cells with puromycin, lentiviral-mediated silencing of AMPK*α*1 in Abcd1-KO mice mixed glial cells was highly successful (Figure 1(b)). Western blot analysis showed complete loss of AMPK*α*1 protein in Abcd1-KO mixed glial cells silenced for AMPK*α*1 (Figure 1(b)(i)). Real-time PCR with primers against mouse AMPK*α*1 showed significant (*P* < 0.001) downregulation of AMPK*α*1 expression (Figure 1(b)(ii)). Silencing with nontargeting scrambled sequence (Scr) had no effect on the levels of AMPK*α*1, thereby suggesting that lentiviral vector-mediated knockdown in Abcd1-KO mixed glial cells was specific for AMPK*α*1.

### 3.2. Mitochondrial Complex (I-V) Gene Expression and Levels Are Reduced in ALD Patient-Derived Cells and in Abcd1-KO Mixed Glial Cells Deleted for AMPK*α*1

**Figure 2:**
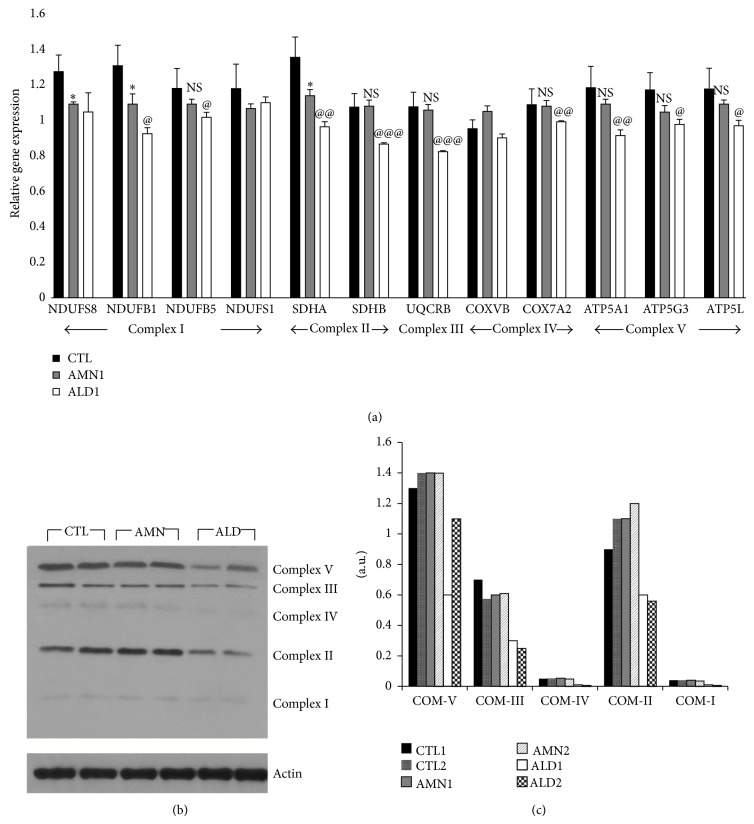
Mitochondrial complex subunit expression and levels in healthy control, adrenomyeloneuropathy (AMN), and adrenoleukodystrophy (ALD) patient-derived fibroblasts. Primary patient-derived skin fibroblasts from healthy control (CTL), AMN, and ALD were cultured as described in Section 2. mRNA (a) and protein (b) levels of mitochondrial complex subunits were significantly reduced in ALD patient-derived fibroblasts. (c) Densitometric ratio of mitochondrial subunit levels versus actin in western blots. Results represent the mean ± SD of triplicates from two different experiments. ^@^ *P* < 0.05, ^@@^ *P* < 0.01, and ^@@@^ *P* < 0.001 ALD compared with AMN. ^*^ *P* < 0.05 AMN compared with CTL. COM: complex, NS: nonsignificant.

There is increasing evidence that mitochondrial dysfunction is important in the pathophysiology of X-ALD [20, 21]; however, the exact upstream mechanism(s) remain hypothetical at this point [22]. AMPK is the principal upstream regulator of mitochondrial biogenesis and function [15]. We documented the first report that AMPK*α*1 levels and mitochondrial function (OCR), a measure of oxidative phosphorylation (OXPHOS), were decreased in ALD patient-derived cells [9]. The OXPHOS system is composed of five complexes, four of which, complexes I-IV, cooperate to generate a proton gradient across mitochondrial inner membrane. Complex V generates the universal energy ATP coupling with proton flow [23]. Quantitative PCR (Figure 2(a)) for individual subunits comprising complexes I-V and antibody cocktail against all five complexes showed (Figure 2(b)) that mitochondrial complex subunit expressions and levels were also significantly decreased in ALD patient-derived cells when compared to AMN patient-derived cells. Two subunits of complex I (NDUFS8 and NDUFB1) and 1 of complex II (SDHA) were significantly (*P* < 0.05) reduced in AMN fibroblasts when compared to control healthy patient-derived fibroblasts, while the rest of the subunit expressions were unchanged between AMN and healthy control patient-derived cells (Figure 2(a)).

**Figure 3:**
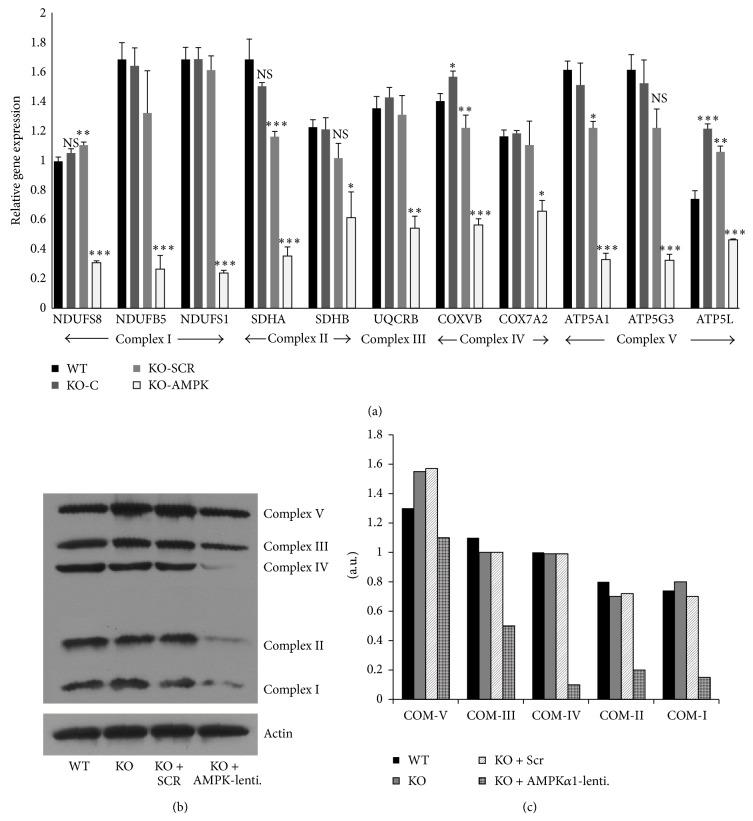
Mitochondrial complex subunit expression and levels in wild type, Abcd1-knockout (KO), and adenosine monophosphate activated protein kinase- (AMPK*α*1-) deleted Abcd1-KO primary mixed glial cells. Wild type (WT) and Abcd1-KO primary mixed glial cells were cultured as described in Section 2. Abcd1-KO mixed glial cells were silenced for scrambled control (Scr) or AMPK*α*1 as described in Section 2. mRNA (a) and protein (b) levels of complex subunits were significantly reduced in Abcd1-KO mixed glial cells deleted for AMPK*α*1. (c) Densitometric ratio of mitochondrial subunit levels versus actin western blots. Results represent the mean ± SD of triplicates from two different experiments. ^*^ *P* < 0.05; ^**^ *P* < 0.01; ^***^ *P* < 0.001. COM: complex, NS: nonsignificant.

Similar to human AMN cells (compared to healthy controls) (Figure 2(a)), mitochondrial complex subunit expressions and levels in Abcd1-KO mice mixed glial cells were comparable to control WT mice mixed glial cells (Figure 3(a)). This could be expected since AMPK*α*1 levels were unchanged between Abcd1-KO mice's central nervous systems and mixed glial cells (Figure 1(b)). Moreover, Abcd1-KO mice mimic human AMN phenotype [8]. This provided us with an opportunity to test if deletion of AMPK*α*1 in Abcd1-KO mixed glial cells could mimic changes in mitochondrial complex expressions similar to that found in ALD patient-derived cells (Figure 2). Abcd1-KO mice mixed glial cells deleted for AMPK*α*1 indeed had significantly reduced expression (Figure 3(a)) and protein levels (Figure 3(b)) of mitochondrial complex subunits. Although their individual role in X-ALD remains to be investigated, underexpression of multiple mitochondrial subunits has been associated with neurodegenerative conditions [24]. For instance, Leigh's syndrome is a severe neurodegenerative disease associated with mutations and underexpression of mitochondrial complexes [25–28]. Mutations and underexpression of multiple complex I subunits (including NDUFS1 and NDUFS8 observed here in ALD fibroblasts/AMPK*α*1-deleted Abcd1-KO cells) are associated with severe Leigh's syndrome [25–27]. Homozygous mutations in the complex II subunit SDHA gene are also associated with Leigh's syndrome [28]. Complex II is critical to mitochondrial function since it lies at the cross-point of OXPHOS and Krebs-cycle pathways. SDHA levels were decreased in ALD patient-derived fibroblasts (Figure 2) as well as in AMPK*α*1-deleted Abcd1-KO mixed glial cells (Figure 3). Despite reduced SDHA expression (Figure 2), AMN patient-derived cells had mitochondrial OCR and ATP levels comparable to healthy controls [9]. This may be attributed to the fact that expression of complex V subunit (ATP-synthase subunit) was not significantly altered between AMN and healthy controls (Figure 2). Complex V subunit expression was decreased in ALD patient-derived cells (Figure 2) and in AMPK*α*1-deleted Abcd1-KO mice mixed glial cells (Figure 3). Deficiency of the complex V subunit ATP5A1 is associated with fatal neonatal encephalopathy [29].

**Figure 4:**
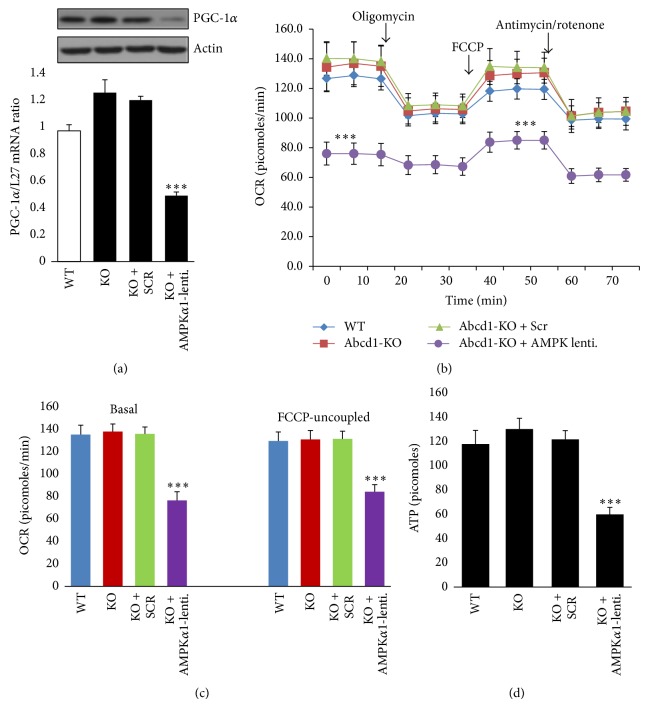
Mitochondrial bioenergetics in adenosine monophosphate activated protein kinase- (AMPK*α*1-) deleted Abcd1-knockout (KO) primary mixed glial cells. (a) Proliferator-activated receptor gamma coactivator 1-alpha (PGC-1*α*) protein and mRNA levels were significantly reduced in Abcd1-KO cells when deleted for AMPK*α*1. (b) Abcd1-KO mice mixed glial cells (1.5 × 10^4^ cells/well) were plated in XF^96^V3-PS cell culture plates (Seahorse Bioscience) and deleted for AMPK*α*1 as described in Section 2. Mitochondrial oxygen consumption rate (OCR) was measured by sequential addition of oligomycin, FCCP, and antimycin/rotenone to measure basal and FCCP-uncoupled OCR. (c) Basal and FCCP-linked OCR was similar between wild type (WT) and Abcd1-KO mixed glial cells but decreased in Abcd1-KO mixed glial cells deleted for AMPK*α*1. (d) A parallel 96-well plate was used for adenosine triphosphate (ATP) measurement. Results represent the mean ± SD of triplicates from three different experiments. ^***^ *P* < 0.001. NS: nonsignificant.

AMPK affects mitochondrial biogenesis principally acting through peroxisome PGC-1*α* [15, 30, 31]. AMPK regulates both the PGC-1*α* activation (phosphorylation) and its transcription [30, 31]. PGC-1*α* in turn drives mitochondrial biogenesis through multiple mitochondrial transcription factors [30, 31]. PGC-1*α* levels were similar between WT and Abcd1-KO mice mixed glial cells (Figure 4(a)). Knockdown of AMPK*α*1 significantly decreased (*P* < 0.001) PGC-1*α* expression and levels in Abcd1-KO mice mixed glial cells (Figure 4(a)). Indeed, PGC-1*α* levels were also decreased* in vivo* in AMPK-KO mice [15]. Whether the underexpression of mitochondrial complex subunits in ALD patient fibroblasts is due to loss of AMPK, mutations in genes encoding the mitochondrial subunits, or a combination of both remains to be investigated.

### 3.3. Mitochondrial OCR Is Comparable between WT and Abcd1-KO Mixed Glial Cells and Is Significantly Reduced in Abcd1-KO Mixed Glial Cells Deleted for AMPK*α*1

We recently documented that mitochondrial OCR, a measure of mitochondrial OXPHOS, was significantly reduced in ALD (but not AMN) patient-derived cells [9]. These ALD patient-derived cells also had a loss of AMPK and decreased levels of mitochondrial complex (I-V) subunit genes and proteins (Figures 2(a)-2(b)). Having demonstrated that AMPK*α*1 levels are similar between WT and Abcd1-KO mice mixed glial cells and that deletion of AMPK*α*1 significantly reduces mitochondrial complex (I-V) subunit expression and protein levels (Figure 3), we next characterized the bioenergetics of these cells using a Seahorse extracellular flux (XF^e^96, Seahorse Bioscience) analyzer. In this system mitochondrial respiration (OCR) is used to measure OXPHOS in intact cells [32]. WT and Abcd1-KO mice mixed glial cells (1.5 × 10^4^/well) were plated in a XF^e^96-well microplate (Seahorse Bioscience). Abcd1-KO cells were deleted for AMPK*α*1 within the 96-well plate and OCR was measured 48 hours after AMPK*α*1 deletion (Figure 4(b)). Basal and FCCP-uncoupled maximal OCR (a measure of mitochondrial integrity [9]) were similar in WT and Abcd1-KO mice mixed glial cells (Figures 4(b) and 4(c)). Scramble (Scr) silencing did not impact the basal and FCCP-uncoupled OCR values between control and Scr silenced Abcd1-KO mixed glial cells (Figures 4(b) and 4(c)). On the other hand, deletion of AMPK*α*1 significantly (*P* < 0.001) reduced both the basal and FCCP-uncoupled OCR levels in Abcd1-KO mixed glial cells (Figures 4(b) and 4(c)). This provides evidence of a direct causal role for AMPK*α*1 in mitochondrial dysfunction in ALD, which was recently postulated by us in ALD patient-derived cells [9].

Mitochondrial OXPHOS generates the ATP energy for the cell [33]. Downregulation of complex V may affect the synthesis of ATP and ATP-dependent processes [33]. Therefore, the decreased expression of complex V subunits in ALD patient-derived cells observed in this study (Figure 2) supports our recent report of decreased ATP levels in the same patient-derived cells [9]. Abcd1-KO mice mimic human AMN phenotype, and primary mixed glial cells from Abcd1-KO mice (compared with WT mixed glial cells) did not exhibit a decrease in ATP levels (Figure 4(d)) similar to our observation in human AMN patient-derived cells [9]. However, deletion of AMPK*α*1 led to a significant decrease (*P* < 0.001) in ATP levels in Abcd1-KO mice mixed glial cells (Figure 4(d)) in line with our observation of decreased complex V levels (Figure 3) and mitochondrial OCR (Figures 4(b) and 4(c)) in these cells. These findings, together with our recent report of differential loss of AMPK*α*1 in ALD [9], indicate a detrimental role for loss of AMPK*α*1 in inducing mitochondrial dysfunction in severe ALD pathology.

### 3.4. Deletion of AMPK*α*1 Induced Spontaneous Proinflammatory Response in Unstimulated Abcd1-KO Mice Mixed Glial Cells

**Figure 5:**
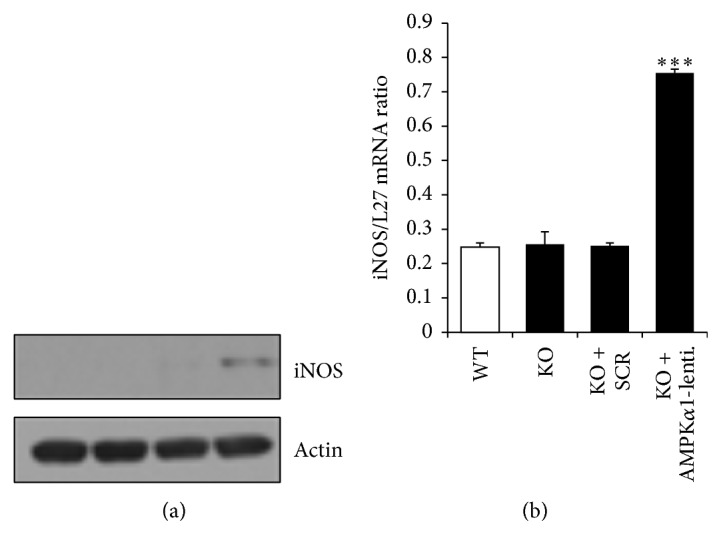
Adenosine monophosphate activated protein kinase- (AMPK*α*1-) deletion induces a spontaneous inflammatory response in Abcd1-KO mixed glial cells. Abcd1-KO mice primary mixed glial cells were plated in 6-well plates and deleted for AMPK*α*1 using lentiviral vector. Inducible nitric oxide synthase (iNOS) protein (a) and mRNA levels (b) were induced in Abcd1-KO mixed glial cells when deleted for AMPK*α*1 using lentiviral particles. Results represent the mean ± SD of triplicates from three different experiments. ^***^ *P* < 0.001.

The mechanism of the neuroinflammatory response in X-ALD remains unknown.* In vitro* loss of peroxisomal transporters (Abcd2 and Abcd3) that have significant homology to the Abcd1 gene has been shown to induce an inflammatory response in central nervous system cells [4]. However, levels of Abcd2 and Abcd3 are unaltered in the central nervous systems of X-ALD patients and, therefore, are likely not the disease modifying genes for initiation and progression of X-ALD [34]. Since AMPK*α*1 levels were reduced only in ALD patient-derived cells that presented with increased expression of proinflammatory genes [9], this provides a causal association between AMPK*α*1 loss and induction of the proinflammatory response in the ALD phenotype. This is expected since AMPK*α*1 is crucial for the anti-inflammatory skewing of cells [10, 19] and is involved in inhibiting lipid-induced inflammation [18]. Furthermore, AMPK-knockout animal models consistently demonstrate increased proinflammatory skewing [35]. To provide a direct evidence for this causal association between AMPK*α*1 loss and development of the proinflammatory response in X-ALD, we investigated the expression and levels of inducible nitric oxide synthase (iNOS) in Abcd1-KO mixed glial cells deleted for AMPK*α*1 (Figure 5).* In vivo* [4] and* in vitro* [16] evidence implicates involvement of iNOS in X-ALD neuropathology. Basal iNOS levels were undetectable in WT and Abcd1-KO mixed glial cells (Figure 5). Silencing with control (Scr) lentiviral particles did not induce any iNOS level (Figure 5). However, AMPK*α*1 deletion significantly induced (*P* < 0.001) the iNOS level (Figure 5(a)) and gene expression (Figure 5(b)) in unstimulated Abcd1-KO mixed glial cells.

## 4. Conclusions

In conclusion, these findings represent the first direct evidence of a link between loss of AMPK*α*1 and initiation/augmentation of mitochondrial dysfunction and the neuroinflammatory response in X-ALD, especially in mixed glial cells. The central nervous system (brain and spinal cord) is the target organ for development of X-ALD therapies. AMPK*α*1, therefore, provides a novel target for development of therapeutic strategies aimed at ameliorating the initiation and/or progression of the neuroinflammatory response in X-ALD.
